# Fifteen-Year Follow-Up: Treatment of Recurrent Mandibular Giant Cell Lesion—From Resection to Rehabilitation

**DOI:** 10.1155/crid/2248326

**Published:** 2025-03-04

**Authors:** Thiago Schneider, Henrique Martins da Silveira, Guilherme Teles, Bruno Dias, Carlos Fernando Mourão

**Affiliations:** ^1^Department of Oral and Maxillofacial Surgery, Craniofacial Anomaly Treatment Center, Rio de Janeiro, Brazil; ^2^Department of Oral and Maxillofacial Surgery, Rio de Janeiro State University, Rio de Janeiro, Brazil; ^3^American Dental Institute, Orlando, Florida, USA; ^4^Department of Clinical and Translational Research, Tufts University School of Dental Medicine, Boston, Massachusetts, USA

**Keywords:** alveolar distraction osteogenesis, autogenous bone grafting, central giant cell granuloma, dental implants, mandibular reconstruction

## Abstract

**Aim:** This case report details the long-term management of a recurrent central giant cell granuloma (CGCG) in the anterior mandibular arch of a 28-year-old female.

**Case Report:** Following initial surgical resection in 2007, which resulted in a significant bony defect, a multidisciplinary approach was employed to restore mandibular integrity and function. In 2009, alveolar distraction osteogenesis was performed using a DePuy Synthes alveolar distractor. Subsequently, in 2011, autogenous onlay bone grafting was carried out to optimize the alveolar ridge contour. Four Neodent CM implants were placed in 2012, followed by prosthetic rehabilitation. The 15-year follow-up in 2024 revealed excellent outcomes, with stable peri-implant tissues, maintained bone levels, and a fully functional prosthesis. This case highlights the efficacy of combining advanced surgical techniques, including distraction osteogenesis and autogenous bone grafting, in managing complex CGCG cases.

**Results and Conclusion:** The successful long-term outcome underscores the importance of a comprehensive treatment approach and regular follow-up in addressing the challenges posed by aggressive and recurrent CGCGs. This report provides valuable insights into the potential for fully functional and aesthetic restoration following extensive CGCG treatment, emphasizing the benefits of a well-coordinated, multidisciplinary approach in maxillofacial reconstruction.

## 1. Introduction

Central giant cell granuloma (CGCG) is a benign but potentially aggressive osteolytic lesion that predominantly affects the jaws. Characterized by multinucleated giant cells within a fibroblastic stroma, CGCG accounts for approximately 7% of all benign tumors in the maxillofacial region [1, 2]. Despite its benign classification, CGCG can lead to significant bone destruction and facial deformity and has a propensity for recurrence, particularly in younger patients under 30 years of age, with a noted female predilection [2].

The etiology of CGCG remains controversial, with proposed factors including trauma, inflammation, intraosseous hemorrhage, and genetic predisposition. This ambiguity in origin contributes to the challenges in its management. CGCGs are typically classified as aggressive or nonaggressive based on their clinical and radiographic features. Aggressive variants are characterized by rapid growth, pain, cortical perforation, root resorption, and a higher recurrence rate, necessitating more intensive treatment approaches [3].

Surgical management has traditionally been the mainstay of CGCG treatment, with options ranging from conservative curettage to radical resection. While curettage offers lower morbidity, it is associated with a higher risk of recurrence, especially in aggressive lesions. Conversely, radical resection provides better tumor control but can result in significant functional and aesthetic deficits. The challenge lies in balancing adequate tumor removal with the preservation of vital structures and minimizing postoperative morbidity, particularly in younger patients.

Recent advances in surgical techniques, including three-dimensional planning and computer-assisted navigation, have improved precision in resection and reconstruction. However, the optimal management strategy for CGCGs, particularly in recurrent or aggressive cases, remains a subject of ongoing debate. Adjunctive pharmacological therapies, such as intralesional corticosteroids [4] or calcitonin [5] nasal spray, have been explored to reduce lesion size or recurrence and potentially avoid extensive surgery, but their long-term efficacy requires further investigation.

This case report is aimed at detailing the long-term management of a recurrent CGCG in a 28-year-old female's anterior mandibular arch. Initial surgical resection was followed by alveolar distraction osteogenesis (ADO) and autogenous bone grafting to restore mandibular integrity. Over a 15-year follow-up, the multidisciplinary approach combining advanced surgical and prosthetic techniques proved effective, highlighting the importance of comprehensive treatment planning and integrating multiple therapeutic modalities for managing complex CGCG cases.

## 2. Case Report

**Figure 1:**
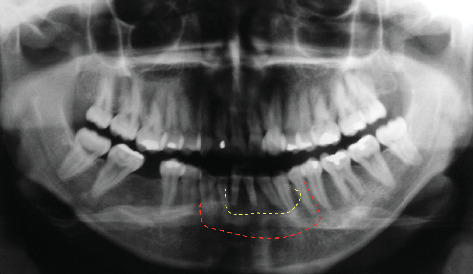
Preoperative panoramic radiograph showing the lesion (yellow dashed line) and planned surgical approach (red dashed line).

The present case report details the long-term management of a recurrent CGCG in a 28-year-old female's anterior mandibular arch (Figure 1). Initial surgical resection was followed by ADO and autogenous bone grafting to restore mandibular integrity. Over a 15-year follow-up, the multidisciplinary approach combining advanced surgical and prosthetic techniques proved effective, highlighting the importance of comprehensive treatment planning and integrating multiple therapeutic modalities for managing complex CGCG cases.

This study was conducted in compliance with ethical guidelines, and written informed consent was obtained from the patient for publication of the case details, including clinical photographs and radiographs. The signed consent form has been securely archived in accordance with institutional policies, and no identifiable patient information is disclosed in the manuscript.

**Figure 2:**
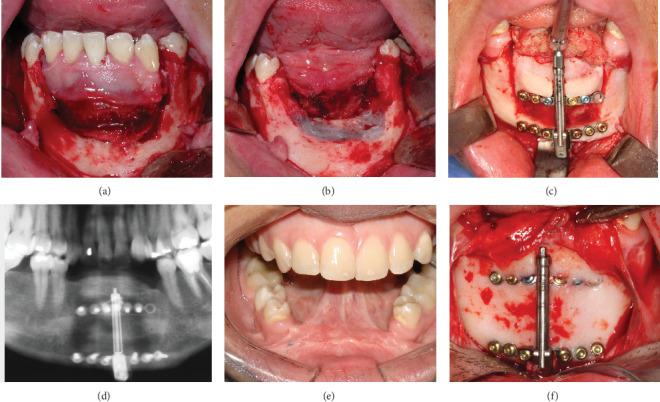
(a) Intraoperative image of the central giant cell granuloma in the anterior mandible. (b) Immediate post-resection image of the tumor. (c) Alveolar distractor in place, showing the distraction osteogenesis process for vertical bone augmentation. (d) Panoramic X-ray 1-year postdistraction. (e) Clinical bone level and mucosal integrity 1 year after the distraction surgery. (f) Clinical image of the surgical procedure to remove the bone distractor.

In 2007, a healthy 28-year-old female patient presented with a medical history of recurrent CGCG in the anterior mandibular arch. Following comprehensive clinical and radiographic evaluation, the initial treatment plan involved surgical resection of the lesion (Figure 2a,b). The procedure resulted in a significant bony defect, compromising the mandible's structural integrity and aesthetic contour, thus necessitating a complex reconstructive approach.

Two years after the resection, in 2009, the surgical team opted for ADO to address the substantial bony deficit. A DePuy Synthes alveolar distractor (Johnson & Johnson, New Brunswick, New Jersey, United States) with a 16-mm distraction capacity was selected for the procedure. Preoperative planning involved detailed radiographic analysis and virtual surgical planning to determine the optimal vector of distraction (Figure 2c).

The distractor was implanted using a transmucosal approach under general anesthesia. Following a latency period of 5–7 days, the distraction protocol was initiated at a rate of 1 mm per day, divided into two 0.5-mm activations. The consolidation phase extended over a period of 1 year to ensure adequate bone maturation and stability (Figures 2d, 2e, and 2f).

**Figure 3:**
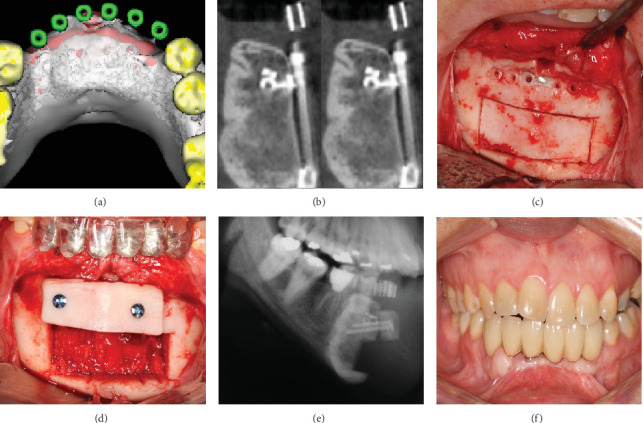
(a) 3D reconstruction planning before implant placement. (b) Autogenous bone graft harvested from the mandibular anterior arch after distractor removal. (c) Bone graft placed near the crestal area for better posterior implant positioning. (d) CBCT image demonstrating lingual bone growth. (e) Cephalometric X-ray immediately after bone grafting. (f) Clinical image of the crowns after the 15-year follow-up.

A cone-beam computed tomography (CBCT) scan performed in late 2010 revealed successful vertical augmentation of the alveolar ridge. However, the scan also demonstrated a slight lingualization of the distraction vector, resulting in suboptimal buccal-lingual dimensions for ideal implant placement (Figure 3a,b).

To address this issue, in 2011, an autogenous onlay bone grafting procedure was performed. Corticocancellous bone blocks were harvested from the lingual aspect of the distracted area and fixed to the buccal surface using titanium microscrews. This technique improved the vestibular contour and enhanced the overall bone volume and quality in the reconstructed area (Figures 3c, 3d, and 3e).

Following a 6-month healing period, in 2012, four Neodent CM implants (Straumann Group, Curitiba, Brasil) measuring 3.5 mm in diameter and 11.5 mm in length were placed in the augmented mandibular arch. Implant stability measurements using the manufacturer's torque wrench indicated excellent primary stability values of 45 to 60 Ncm for all implants. After 4 months of bone healing/osseointegration, a screw-retained fixed partial denture was fabricated and delivered, restoring both function and aesthetics.

Clinical and radiographic examinations were conducted in 2024 as part of a long-term follow-up protocol. Intraoral photographs and a digital panoramic radiograph were obtained to assess the stability of both hard and soft tissues around the implants and the integrity of the prosthetic rehabilitation.

The 15-year follow-up revealed outstanding outcomes (Figure 3f). The peri-implant soft tissues clinically demonstrated optimal health with no signs of inflammation or recession. Radiographically, all four implants exhibited stable crestal bone levels with no evidence of progressive bone loss or peri-implantitis. The prosthetic superstructure remained fully functional, with no mechanical complications such as screw loosening or porcelain chipping observed.

## 3. Discussion

This case report illustrates the successful long-term management of a recurring CGCG in the anterior mandibular arch through a comprehensive, multidisciplinary approach. The initial surgical resection, which resulted in a substantial bone defect, was effectively addressed using two different reconstructive techniques: ADO followed by autogenous bone grafting. This combination of procedures proved instrumental in restoring both the functional and aesthetic aspects of the mandible.

The 15-year follow-up period demonstrated remarkable stability and durability of the reconstructed mandible. Regular clinical and radiographic evaluations confirmed the absence of recurrence and the maintenance of both hard and soft tissue integrity around the implants. The patient's reported satisfaction with functional and aesthetic outcomes further validates the efficacy of the employed treatment strategy.

The recurrence of CGCG observed in this case underscores a well-documented challenge in managing these lesions. Literature reports recurrence rates varying from 11% to 49%, with higher rates typically associated with aggressive variants and younger patients [2]. The initial recurrence in this patient, despite conservative surgical intervention, emphasizes the critical importance of long-term follow-up and the potential necessity for more comprehensive treatment strategies in CGCG management. In the present case report, the patient tried to use intralesional corticosteroids to reduce the tumor's size. However, it was not effective in her case.

Implanting the ADO in a second-stage surgery was essential after an aggressive resection to address substantial bone defects resulting from CGCG resection. While ADO is widely utilized in posttraumatic and congenital defects, its use in post-CGCG reconstruction is less frequently reported [6, 7]. The successful outcome, in this case, suggests that ADO, particularly when combined with subsequent autogenous bone grafting, can be an effective technique for restoring both form and function in patients with large mandibular defects following CGCG resection.

The long-term stability of the reconstruction and the absence of recurrence over a 15-year period in this case are particularly noteworthy. This outcome strongly supports the efficacy of the combined surgical approach employed. However, it is crucial to acknowledge that the literature demonstrates variability in the long-term outcomes for CGCG treatment. Previous studies have shown that recurrence can occur even after extended periods, with some cases reported more than 5 years posttreatment [2, 8]. This underscores the necessity for prolonged follow-up in CGCG cases, regardless of the initial treatment modality.

The successful rehabilitation with dental implants, in this case, demonstrates the potential for full functional restoration following extensive CGCG treatment. The stability of peri-implant tissues and bone levels over the 15-year follow-up period is particularly encouraging. These findings align with other case reports and small series that have shown successful implant-based rehabilitation in reconstructed jaws following CGCG treatment. However, it is important to emphasize that achieving such positive outcomes in complex cases of recurring CGCG requires careful case selection, meticulous surgical planning, and regular long-term monitoring.

## 4. Conclusion

This case also underscores the importance of a well-coordinated, multidisciplinary approach in managing complex maxillofacial defects caused by aggressive CGCG. The combination of precise surgical interventions and meticulous prosthetic rehabilitation can achieve long-term success and patient satisfaction. Future cases can benefit from the lessons learned in this report, emphasizing the need for innovative and integrated treatment modalities to address the challenges posed by recurrent CGCG.
